# Daytime Grazing in Mountainous Areas Increases Unsaturated Fatty Acids and Decreases Cortisol in the Milk of Holstein Dairy Cows

**DOI:** 10.3390/ani11113122

**Published:** 2021-10-31

**Authors:** Jalil Ghassemi Nejad, Bae-Hun Lee, Ji-Yung Kim, Kyung-Il Sung, Hong-Gu Lee

**Affiliations:** 1 Department of Animal Science and Technology, Konkuk University, Seoul 05029, Korea; jalilgh@konkuk.ac.kr; 2 National Institute of Animal Science, RDA, Cheonan 31000, Korea; leebaehun@korea.kr; 3 College of Animal Life Sciences, Kangwon National University, Chucnheon 24341, Korea; oversboy02@kangwon.ac.kr (J.-Y.K.); kisung@kangwon.ac.kr (K.-I.S.)

**Keywords:** milk cortisol, milk fatty acids, grazing animals, Holstein dairy cows, omega-3 fatty acids

## Abstract

**Simple Summary:**

The effects of grazing dairy cows in mountainous areas for 12 and 24 h compared with keeping the cows under confined settings were evaluated by examining the overall levels of fatty acids and cortisol in milk as an indicator of stress. Our findings revealed favorable changes in cows grazing for 12 h, with significantly improved milk fatty acid profile and decreased milk cortisol content. Additionally, we found no significant changes in the milk yield of 12 h grazed cows compared to the control group of confined cows, while milk fat and protein showed higher values in 12 h grazed cows. Grazing for 24 h caused a significant decrease in the milk yield compared to the two other groups. Overall, grazing for 12 h in a mountainous area is suggested to be beneficial for animal welfare, with positive improvements in milk fatty acids and lessening of stress levels without adverse effects on milk yield.

**Abstract:**

The effects of grazing lactating cows in mountainous areas for 12 and 24 h compared with the confined indoor system were evaluated by examining the overall milk fatty acid and cortisol. Twenty-one dairy cows were allocated to three treatment groups: (1) control (confined management system in a free-stall barn; TMR based), (2) grazing for 12 h (12 hG; TMR plus grazing pasture), and (3) grazing for 24 h (24 hG; pasture-based feeding system). Dry matter intake was higher in the control and 12 hG groups than in the 24 hG group. The yields of total milk and the 3.5% fat-corrected milk were the lowest in the 24 hG group. Milk fat was the highest in the 24 hG group and higher in 12 hG compared with the control group. Milk protein and lactose levels were the highest in the 12 hG group. The highest somatic cell count was observed in the 24 hG group. The saturated fatty acid levels were higher in the control group compared with the 12 hG and 24 hG groups. There was no difference in overall mono-unsaturated fatty acids between 12 hG and 24 hG groups. Poly-unsaturated fatty acids were higher in the 12 hG group compared with the control and 24 hG groups. There was no difference in omega-6 (ω-6) fatty acids among the groups, and omega-3 fatty acids were higher in the 12 hG group than in the control group. Milk cortisol was the highest in the 24 hG group and higher in the control group compared with the 12 hG group. Taken together, grazing for 12 h is advisable for farms that have access to mountainous areas to improve the milk fatty acid profile and decrease the stress levels in high-yielding Holstein lactating cows.

## 1. Introduction

Grazing is an important management practice in terms of, for example, the nutrition of fresh forage intake, animal health [1], animal well-being, natural behavior [2], landscape values, and grassland biodiversity [1,3,4]. Grazing dairy cows has been practiced for a long time as a strategy for improving animal welfare and milk quality [5], lessening oxidative stress [2], and economic purposes [6,7].

One indicator to measure animal welfare is cortisol concentrations in different matrices, including milk. Measuring cortisol in milk is becoming a promising indicator for evaluating the daily or short-term stress conditions and well-being of dairy animals [8,9] and humans [10]. Measuring plasma cortisol as a primary stress indicator is problematic because the animal needs to be restrained for invasive blood collection [11,12], and measuring cortisol in milk has been suggested as alternative due to its accessibility regardless of the management system together with the ease of collecting samples in a manner that does not cause stress [9]. Therefore, to support the hypothesis on the potential of grazing animals in decreasing stress, particularly in dairy cows [7], we further tested the hypothesis of whether grazing cows have decreased levels of milk cortisol compared with indoor animals in this study.

Korea is one of the countries that need to improve land use efficiency because it has only 16% arable land [7] that can potentially be used for agricultural and livestock purposes using smart farming. It should be noted that Korea imports 75% of its compound feed and 96.4% of its feed crops, which has become a matter of concern among Korean livestock industry participants and the Korean government [12,13]. In addition to its economic advantages (free pasture, decreasing cost of human resources, etc.), grazing pasture allowance can reduce the negative effects of high stock density faced by farms of the cow industry in Korea. Given that 70–80% of Korea is mountainous [7], the application of smart farming is necessary for using the accessible land beside livestock farms with controlled grazing using electric fences for containment.

Many factors influence the compositions of milk including but not limited to the nutritional values of feed, nutrition level, feeding management, stress source and severity, genetic, environment, season, stage of lactation, reproductive and productive status of the animals, age, and BW of the animals, etc. Improving milk quality has the dual purpose of increasing both the nutritional value and the commercial value of the milk (i.e., low worker and feed costs). In this regard, increasing the levels of poly-unsaturated fatty acids (PUFAs), particularly omega-3 PUFA, in milk is desirable in dairy animals [1,6]. Although previous studies have investigated the effects of grazing on the welfare [4,14,15] and milk of dairy animals [16,17], to what extent grazing can positively impact milk characteristics without negatively influencing production performance in high-yielding lactating cows needs further exploration. Therefore, this study was designed to evaluate the effect of allowing daytime grazing for 12 h and full-time grazing of 24 h compared with the indoor rearing of animals on the milk yield and characteristics, such as milk unsaturated fatty acid and cortisol contents, in high-yielding lactating cows.

## 2. Materials and Methods

All experimental procedures involving animals were approved by the Animal Welfare and Ethics Committee of Kangwon National University, Chuncheon, Republic of Korea (KIACUC-16–0098). This study was carried out in spring at the Sky Ranch farm (Daegwallyeong, Pyeongchang, Gangwon Province, Korea) 920 m above sea level.

### 2.1. Experimental Animals and Treatment Application

Twenty-one dairy cows (age = 36 ± 4.5 months) were used in this study. The specifications of BW, milk yield, and parity of the experimental animals are presented in Table 1.

The control group (indoor rearing) was fed total mixed ration (TMR) plus additional commercial concentrate feed during milking. The maintenance system was indoor (confined management system). The TMR was consisted of forage (alfalfa hay, timothy hay, tall fescue straw, oat straw), silage (reed canary grass silage), concentrate feed (commercial concentrate), ad vitamin-mineral mix plus protected fat. The ratio of forage to concentrate was 42:58. The indoor facility was equipped with free stalls bedded with river dry sand. The pasture encompassed a mixture of timothy and reed canary grass. The pastureland was cultivated in 2014 and planted by timothy grass for the purpose of using the land for grazing cows. Electric fences were used to divide paddocks, and grazing was allowed when the grass was approximately 30 cm tall. TMR samples were collected for composition analysis at three times during the study: the beginning, middle, and end. The chemical compositions of the TMR and commercial concentrate feed that were used are provided in Table 2. Water was provided using a water trough in the barn for the control group and using connecting hoses in the pasture for the grazing cows.

The grazing cows were divided into two groups: the 12 h grazing (12 hG) and 24 h grazing (24 hG) groups. The 12 hG group was not only fed TMR while indoors (18:00–06:00 h) but also allowed to graze during the daytime (06:00–18:00 h). The maintenance system for 12 hG group was half indoor (nighttime; the same as the control, and half (daytime) outdoor grazing, while the 24 hG group was outdoor grazing except the time of milking. A rotational grazing system was applied using four different areas adjacent to the farm each delineated by electric fences. Pasture sampling was conducted biweekly (total 4 times) to analyze chemical compositions. The experiment lasted 5 weeks (mid-late spring; 15 May, 22 June), of which the first week was used for grazing adaptation and conduction and the remaining 4 weeks for the main experiment. For adaptation, grazing animals were grazed daily for 3 h, increasing the time of grazing to meet the grouping criteria. The chemical compositions of the pastures used by the 12 hG and 24 hG groups are illustrated in Table 3.

Before starting the actual experiment, the animals underwent 7 days of training to graze in the pastures after morning milking. For the study of feed intake, the amount of grass before and after grazing was investigated on weekly basis and the difference was calculated as the intake.

### 2.2. Analyses

**Figure 1:**
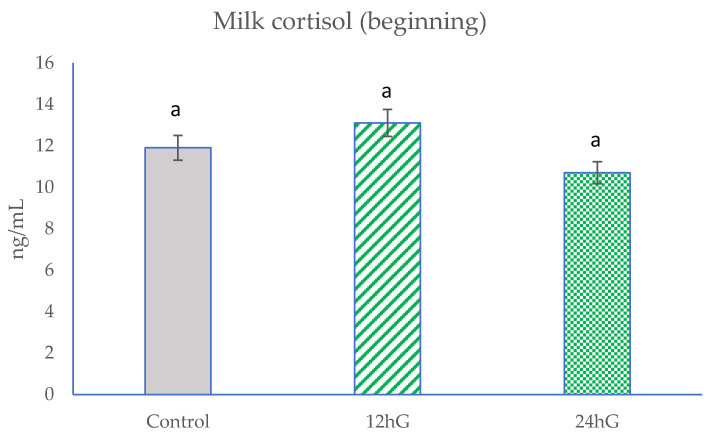
Milk cortisol levels at the beginning of the experiment in dairy cows. Control: non-grazing group; 12 hG: 12 h grazing group; 24 hG: 24 h grazing group. Columns represented by the same letters are not significantly different at *p* > 0.05.

**Figure 2:**
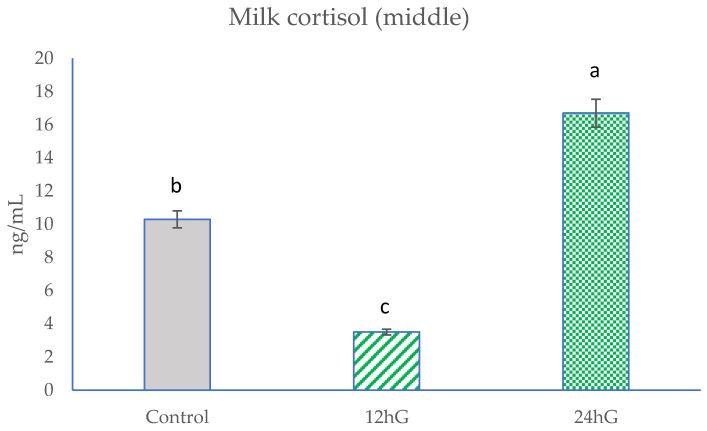
Milk cortisol levels at the middle of the experiment in dairy cows. Control: non-grazing group; 12 hG: 12 h grazing group; 24 hG: 24 h grazing group. Columns represented by the different letters are significantly different at *p* < 0.05.

**Figure 3:**
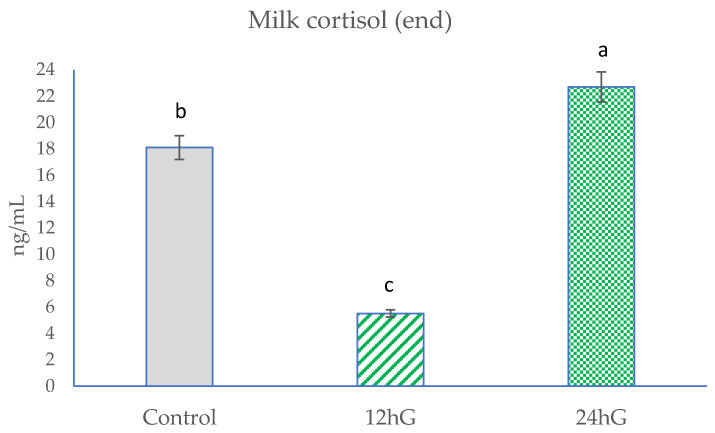
Milk cortisol levels at the end of the experiment in dairy cows. Control: non-grazing group; 12 hG: 12 h grazing group; 24 hG: 24 h grazing group. Columns represented by the different letters are significantly different at *p* < 0.05.

Feed analyses included chemical compositions of TMR (Table 2) and pastures (Table 3). The analyses included milk yield, milk composition (Table 4), milk FAs (Table 5), and milk cortisol (Figures 1–3).

#### 2.2.1. Feed Samples and Analysis

The feed included TMR and pastures. A TMR sample (approximately 1 kg) was collected from the manger at the beginning, middle, and end of the experiment, and the pasture collection method [7] was performed four times (weekly, samples 1 to 4). The pasture sampling was conducted in 5 places in each paddock using 1 m × 1 m quadrat. The samples were stored for chemical analysis. The average chemical compositions of TMR samples are reported because there were no variations in the TMR ingredients. However, we decided to report the chemical composition of the pasture for each sampling time separately as the contents of pasture may possibly differ at each time point. The pasture and TMR samples were dried at 65 °C for 72 h, milled, and stored before further analysis. The chemical compositions of TMR and pastures were measured using the method of the AOAC (1990).

#### 2.2.2. Milk Collection and Analyses

The experiment lasted 5 weeks, and all the cows in this study (control and grazing groups) were milked twice a day, at 06:00 and 18:00 h, using robotic milking machines (VMSTM V300; DeLaval International AB, Tumba, Sweden). Milk samples for component and cortisol analyses were collected at the beginning (15–17 May; for homogenized grouping purpose only prior to the start of the experiment), middle (third week, 5–7 June), and end (fifth week, 19–21 June) of the experiment. In doing so, and to obtain the highest possible accuracy, we collected the milk on 3 consecutive days at each time point to make sure that daily variations in milk components did not affect the outcome of the measures. We then pooled the morning and evening milk samples for all the consecutive days and made one sample for each animal to measure the milk composition, fatty acid profile, and cortisol content.

#### 2.2.3. Milk Composition

Morning and evening milk samples (15 mL) from each cow were collected in 45 mL sterile tubes (Wisd, Seoul, Korea) at each milking time for 3 consecutive days during the experimental period. The pooled composited milk samples were analyzed for concentrations of fat and protein, lactose percentages, and somatic cell count using a Foss 4000 milko Scan (Foss Electronic, Hilleroed, Denmark).

#### 2.2.4. Fatty Acid Profiles of Milk Samples

The fatty acid profiles of the milk samples were analyzed using an Agilent Intelligent Gas Chromatography (GC) system (7890B FID GC, Agilent Technologies, Santa Clara, CA, USA). In brief, for fat extraction of milk samples, the sample was first homogenized. Then, a test solution was prepared to check for interference with undecanoic acid used as an internal standard. We accurately weighed the homogenized sample such that it contained about 100 to 200 mg of fat, placed it in a Mojonnier tube, added about 100 mg of pyrogallol, and then added 2 mL of internal standard solution. Then, we boiled the Mojonnier tube, added 2 mL of ethanol, and waited until the entire sample was thoroughly mixed. Afterward, we added 4 mL of water, stirred well, added 2 mL of ammonium hydroxide, stirred, and sealed the cap of the Mojonnier tube with a rubber band or Teflon tape for 10 min. We mixed the sample with a stirrer every 5 min so that the particles on the wall of the Mojonnier tube could be thoroughly mixed in. After sample decomposition, we added a few drops of phenolphthalein solution and then ammonium hydroxide to maintain the basic (pink) color. Finally, the lower part of the Mojonnier tube was filled with ethanol to allow for easy separation during ether extraction. Saturated fatty acids (SFAs), mono-unsaturated fatty acids (MUFAs), and poly-unsaturated (PUFAs) fatty acids were analyzed by calculating the sum of individual fatty acids and the sum of representative fatty acids.

#### 2.2.5. Milk Cortisol Analysis

The same milk samples that were used for milk composition and fatty acid analyses were subsampled and used for cortisol analysis using the Bovine Cortisol ELISA kit (cat. no: MBS028594, MyBioSource, Inc. San Diego, CA, USA). After subsampling the milk collection, the samples were centrifugated at 1000× *g* (or 3000 rpm) for approximately 20 min. Then, the supernatant was separately and carefully collected from each sample. The assay was either conducted immediately or the sample stored at −20 or −80 °C for later analysis. Both the intra-assay coefficient of variation (CV = 7.9%) and the inter-assay CV (5.2%) were less than 15% (CV% = SD/mean × 100). All the CV% values were compared using concentration and not optical density (OD) values.

### 2.3. Statistical Analysis

Feed intake and milk yield were analyzed by repeated-measures analysis using the MIXED procedure of SAS (ver. 9.01; SAS institute Inc. Cary, NC, USA), and the mean values were compared for significance using Tukey’s *t*-test at *p* < 0.05. The initial values of the days in milk, milk yield. body weight, and the parity were tested as covariate and since they were not significant at the beginning of the study (Table 1), we did not use them as covariates. We have tested several variance and covariance assumption structures including AR(1), UN, CS, ANTE(1), TOEPH, and ARH(1), and the covariance structure that resulted in the lowest values of the Akaike information criterion (AIC), herein AR(1), was chosen for the final analysis as it was the best fit to our design. The least-squares means of each group were calculated, and the differences between means were tested using the PDIFF option with Tukey’s adjustment. The data of non-repeated measures were analyzed using the GLM procedure of SAS. The mean comparisons between the groups were evaluated using Tukey’s test. Different levels of significance were declared as significant at *p* < 0.05, highly significant at *p* < 0.01, and very highly significant at *p* < 0.001.

## 3. Results

### 3.1. Dry Matter Intake, Milk Yield, and Milk Composition

The dry matter (DM) intake was significantly higher (*p* < 0.05) in the control group and 12 hG groups compared with the 24 hG group, whereas no difference (*p* > 0.05) was observed between the control and 12 hG groups. The total milk and the 3.5% fat-corrected milk (FCM) yields were the lowest (*p* < 0.01) in the 24 hG group, while no difference (*p* > 0.05) was observed between the control and 12 hG groups (Table 4).

Milk fat levels were the highest in the 24 hG group and higher in the 12 hG group compared with the control group (*p* < 0.001). Milk protein levels were higher in the 12 hG and control groups compared with the 24 hG group (*p* < 0.01). There was no significant difference in milk protein levels between the control and the 12 hG group (*p* > 0.05). Lactose levels were the highest in the 12 hG and higher in the control group compared with the 24 hG group (*p* < 0.05). The highest somatic cell count (SCC; *p* < 0.05) was observed in the 24 hG group, while the lowest (*p* < 0.05) SCC was in the control group (Table 4).

### 3.2. Milk Fatty Acids

Saturated fatty acid (SFA) levels were higher (*p* < 0.05) in the control group compared with the 12 hG and 24 hG groups, while no difference between the 24 hG and 12 hG groups was observed (*p* > 0.05). There was no difference (*p* > 0.05) in mono-unsaturated fatty acid (MUFA) levels between the 12 hG and 24 hG groups, while MUFA levels were the lowest (*p* < 0.05) in the control group. Poly-unsaturated fatty acid (PUFA) levels were higher in the 12 hG group compared with the control and 24 hG groups, whereas no difference was observed between the 24 hG and control groups (*p* < 0.05). Total UFA levels were the highest in the 12 hG group, while they were higher in the 24 hG group compared with the control group (*p* < 0.05). The SFA/UFA ratio was the highest (*p* < 0.01) in the control group, while no significant difference (*p* > 0.05) was observed between the 12 hG and 24 hG groups (Table 5).

Levels of omega-6 (ω-6) fatty acid, including γ-linolenic acid and arachidonic acid, were not significantly different (*p* > 0.05) among the groups, while linoleic acid levels were significantly higher (*p* < 0.05) in the 12 hG and 24 hG groups compared with the control group. α-Linolenic acid levels were the highest (*p* < 0.001) in the 12 hG group, whereas no significant difference (*p* > 0.05) was observed between the control and 24 hG groups. Eicosapentaenoic and docosahexaenoic acids levels were higher (*p* < 0.01) in the 12 hG group compared with the control group, while there was no difference (*p* > 0.05) between the 12 hG and 24 hG groups and between the control and 24 hG groups. Omega-3 (ω-3) fatty acid content was the highest in the 12 hG group, whereas no significant difference was observed between the control and 24 hG groups (Table 5).

At the beginning of the study, there were no significant differences (*p* < 0.05) in milk cortisol levels among the groups (Figure 1). In the middle of the experiment, milk cortisol levels were the highest (*p* < 0.05) in the 24 hG group and higher (*p* < 0.05) in the control group compared with the 12 hG group (Figure 2). At the end of study, milk cortisol levels were lower (*p* < 0.05) in the 12 hG group compared with the control and 24 hG groups and lower (*p* < 0.05) in the control group compared with the 24 hG group (Figure 3).

## 4. Discussion

Independently of the type of grazing management (strip or rotational), when using high-quality pastures, the pasture use per unit area and pasture intake per cow are major factors determining milk production in grazing dairy cows, both being primarily controlled by pasture allowance [18,19,20]. Several studies have reported increased DMI due to the balanced protein and dietary energy of TMR, which increases the net energy supply and thus milk yield compared with the DMI in the grazing pasture system [21,22,23,24]. Consistent with the abovementioned studies, in this study, the decrease in the dry matter intake (DMI) in the 24 hG group could simply be explained by the fact that this group did not have access to TMR unlike the control (TMR based) and 12 hG (in addition to pasture) groups, which had access to TMR. The reason for no difference in the DMI between the control and 12 hG groups could be attributed to the access of the cows in the 12 hG group to TMR during the 12 h of non-grazing (indoor). The significant decrease in the DMI of the 24 hG group was reflected in the drastic decrease in the total milk yield and the 3.5% FCM compared with the other groups. The decrease in the milk production of the 24 hG group to approximately 20 kg/day indicates that the actual grazing grass intake did not meet the expected grazing grass intake and the energy–protein balance requirements needed for milk production. Méndez et al. [25] and Kolver and Muller [26] have stated that pastures, in general, have relatively lower DM and metabolizable energy (ME) content, resulting in lower DMI and milk production in high or high-medium dairy cows. Additionally, the growth rates of pastures and the variations in the environment could act as a limiting factor in meeting the DM and energy requirements for grazing cows [27]. Hence, the lower DMI and consequently lower milk yield in the 24 hG group observed in this study were arguably expected. Another reason for the lower DMI and consequently lower milk yield of dairy cows in the 24 hG group could be attributed to the higher content of NDF and ADF and lower CP in pastures (Table 3) compared to TMR (Table 2) provided for the other groups. It should be noted that all cows were homogenized when grouping and had no significant difference in milk yield at the beginning of the study (average 32.5 ± 1.35 kg/day). This result is corroborated by similar studies that have reported lower milk yield in the pasture-based feeding system compared with the confined indoor feeding system [21,26,28]. Overall, it is assumed that nutritional management, such as providing TMR for 24 h grazing, should be considered while maintaining grazing pastures to reduce factors that cause a sudden decrease in the production of grazing cows. The amount of grass intake fluctuates greatly and, thus, the management method must be improved. Consistent with these results in the 12 hG group compared with the control group, Di Grigoli et al. [2] demonstrated that pasture grazing and short daily grazing can cause comparable milk production compared to cows permanently kept indoors. A similar phenomenon was observed by Chapinal et al. [29] and Kennedy et al. [30] when comparing grazing cows with confined cows. Contrarily, some studies have reported higher milk yield in free-stalled or confined cows compared with grazing cows [31,32].

Characteristics of the basal diet, including the TMR, concentrate allowance, forage type, forage-to-concentrate (F:C) ratio, fifiber content, and starch content, have a profound influence on fatty acid synthesis in the rumen and, consequently, on the milk fat level and fatty acid profile [1]. Higher milk fat in the 24 hG group compared with the other groups and higher milk fat in the 12 hG group compared with the control group could be attributed to the higher pasture intake having a higher F:C ratio in the 24 hG group compared with the 12 hG group and having access to a pasture resulting in higher fresh fiber intake in both 12 hG and 24 hG groups compared with the control group. The reason for higher milk fat in the 12 hG and 24 hG groups is speculated to be higher acetic acid production in the rumen of grazing cows due to higher fiber intake. Bath [33] revealed that ration with a higher fiber fraction induces the production of acetate as a main building block of milk fat. Thus, as the time of grazing increases, we expect higher milk fat, which is consistent with the obtained result. This result is also in line with a report [2] that showed higher fat content in the milk of daily grazed cows. Protein synthesis requires constituents of the protein synthesis machinery as well as the adequate amino acid availability and a large energy supply [34,35]. The high energy requirement of protein synthesis and turnover is evidenced not only by the reduction in protein synthesis and ion transport [34,35] but also by the decrease in overall protein synthesis as a consequence of caloric restriction [34,35]. The lower milk protein content in the 24 hG group compared with the 12 hG and control groups is likely attributed to the lower protein intake induced by the lower DMI of the corresponding cows on the pasture-based ration compared to those having the balanced protein energy intake of TMR (control) or TMR plus pasture (12 hG). In line with this result, there have been reports showing increased protein in the milk of cows under TMR feeding systems [36,37]. By contrast, Couvreur et al. [38] revealed an increase in milk protein with increased pasture grazing among cows [5]. Lactose, as the main carbohydrate in bovine milk, can be affected by udder health and the energy balance of the diet [39]. Lactose is basically synthesized from glucose that diffuses from blood into mammary epithelial cells. Thus, a ration with balanced energy–protein has a higher chance of producing more lactose compared with a ration with imbalanced energy–protein or is low in either energy or protein [39]. It can be postulated that in this study, the latter scenario was the case for the 24 hG group compared with the other groups. Fox et al. [40] demonstrated that the amount of water diffusing into the alveoli is determined by lactose [39], and as soon as lactose is synthesized, it is packed into secretory vesicles. In fact, the uptake of the lactose precursor (glucose) directly affects milk synthesis [41]. Having said that, the higher milk lactose in the control and 12 hG groups compared with the 24 hG group could be explained by the abovementioned reason. Moreover, Antanaitis et al., [42] have reported that the increase in milk lactose has a strong negative linear relationship with SCC. They underlined the importance of milk lactose as an indicator of subclinical mastitis showing that the increase in lactose levels in cow’s milk was most closely associated with a decrease in the prevalence of subclinical mastitis pathogens such as *S. agalactiae*, *S. aureus* and other *Streptococci*. Given this finding, our result indicated that cows in 24 hG group had higher SCC coinciding with lower milk lactose (and observation of mastitis in this group) compared with the other groups. The relationship between subclinical mastitis, lactose, and grazing pasture allowance should be further studied. However, O’Callaghan et al. [5] did not find any difference in the lactose concentrations of milk from groups under various feeding systems. They found lower milk lactose levels in cows during late lactation and reported higher milk lactose levels during early lactation. The SCC in grazing cows was significantly higher compared with the control group. The higher SCC in grazing cows could be due to mastitis. Another reason for a higher SCC in the 24 hG group could be the wet pasture on many of the days the cows rested in the area. It was observed that the udder area of cows in the 24 hG group was dirtier compared to the control and 12 hG groups, which could explain the higher SCC and incidence of mastitis in the 12 hG group compared with the control group. These results are in contrast with the report by Di Grigoli et al. [2], who found a lower SCC in the milk of grazing cows compared with cows kept in free-stall barns [28,31]. It is worth noting that their cows grazed for a short time (5 h/day). In this regard, the management quality of grazing cows and the environment (i.e., rain) during grazing could be influential factors that need to be considered when interpreting the results of different studies. Another reason for the lower SCC in short-term grazing cows in the abovementioned studies was the lower incidence of subclinical mastitis and greater hygiene of the udder. In this study, the higher SCC in grazing groups was considered a negative effect of grazing compared with the confined indoor system. However, can be prevented through higher quality management.

Milk fatty acids originate from two sources: (a) blood lipids (approximately 60%) intermediated by non-esterified fatty acid (NEFA) or triglyceride-rich lipoproteins and (b) de novo (C_4_–C_16_) synthesis from acetate and butyrate in the mammary glands [43,44]. Di Grigoli et al. [2] indicated the beneficial effect of grazing on the quality of milk, by inducing unsaturated fatty acids (FA), including ω-3 fatty acids [2,45], which eventually improve the health of humans as the final consumer. O’Callaghan et al. [5], in corroboration with Baltušnikienė et al. [46], revealed that pasture-fed cows produce lower saturated fatty acids (SFAs) compared with cows fed TMR, which supports the findings of our study. Lower SFA levels would be more beneficial for human health because of the potential adverse effects of higher SFA levels for humans, such as cardiac issues [47,48]. The health benefits of milk with higher unsaturated fatty acids (UFAs), including mono- and poly-UFAs, have been previously discussed and established [2,5]. A PUFA is a biological membrane structural material, and as a precursor of prostaglandin, it is involved in maintaining homeostasis of the living body, such as through its roles in blood coagulation and various allergic reactions. In line with a previous study [2], in this study, we found that the feeding system influences milk fatty acid levels. By contrast, Di Grigoli et al. [2] reported higher SFA levels in cows grazed for a short time (5 h/day). They speculated that the short time of daily grazing could be a reason for this phenomenon, in which grazing cows are unable to ingest a sufficient mass of green pasture. Consequently, the SFA/UFA ratio decreased in grazing groups compared with the control group due to lower SFA and higher UFA levels in the corresponding groups. Elgersma et al. [1] explained the mechanism behind the higher UFA (MUFA and PUFA and total UFA) levels in grazing grass-fed cows compared with TMR-fed cows that resulted in higher SFA levels in milk fat. Elgersma et al. [1] documented that pasture allowance can induce the production of α-linolenic acid in milk fat. Consistently, we found higher α-linolenic acid levels in the 12 hG group, whereas the reason for the lower α-linolenic acid levels in the 24 hG group remained unclear. Grassland species diversity may improve PUFA transfer efficiency from feed to milk [1]. Because grazed grass is often younger than the forage ingredients of TMR and also because grazing animals prefer leaf blades to stems [1], these fifindings imply that the fatty acid intake and the proportional intake of α-linolenic acid is higher for cows in grazing pastures compared with confined cows. Green pastures are particularly rich in α-linolenic acid. A higher amount of α-linolenic acid in the milk can therefore be attributed to the higher intake of pasture in grazing cows [1], herein the 12 hG group. The lower ω-3 contents of milk in the 24 hG group could be explained by the lower intake of the group, as discussed earlier, and the imbalanced nutritional value of the intake. Omega-6 encompasses linoleic acid, γ-linolenic acid, and arachidonic acid, whereas omega-3 derivatives consist of α-linolenic acid, eicosapentaenoic acid (EPA), and docosahexaenoic acid (DHA). EPA and DHA have several health benefits for humans and, thus, increasing their levels and that of α-linolenic acid is desirable in the milk of dairy cows, resulting in healthier consumers. Moreover, the ratio of ω-3 to ω-6 FA in dietary products is as important as the dietary proportions of SFAs, MUFAs, PUFAs, and total fat [49]. Similar to this study, Di Grigoli et al. [2] also found higher concentrations of EPA and DHA in the milk of grazing cows compared to the free-stall building system. Lower total ω-3 in the 24 hG group compared with the 12 hG group could be attributed to the important ω-3 components, α-linolenic acid, and lower numerical values of EPA and DHA in the corresponding group. Some of the fatty acid results reported in this study are not in line with those of previous studies when comparing the confined management feeding system and the pasture-based grazing system. Some of the factors that could influence the profiles and values of the fatty acids and explain these discrepancies are (1) time allowance for grazing; (2) quality and maturity of the pasture and TMR quality; (3) duration of grazing; (4) physiological status of grazing cows (lactation phase, parity, DIM, etc.); (5) milking interval (two or three times a day) and method (automatic, semi-automatic); (6) animal breed; (7) farm management quality, including the use of smart farming systems and water-providing systems; and (8) environmental and seasonal factors. All these factors should be considered when interpreting the results.

Cortisol is a primary hormone that is released into the blood not only in response to stress stimuli [12,50] but also in response to decreased feed intake and impaired animal behavior [51,52]. The process includes hypothalamic–pituitary–adrenocortical axis (HPA) activation, which is paramount to the physiological endocrine response of the cows to stress. Any change in blood cortisol levels can be reflected by a similar pattern of change in milk since cortisol diffuses into the milk via blood. Milk sample collection is also compatible with animal welfare protocols and would thus be a good approach to measure stress levels [53]. Thus, among the various stress matrices and because of the limitations associated with blood collection (mainly invasive sample collection), milk is considered a preferential matrix in dairy cows to indicate short-term stimulation of the HPA axis, due to the ease of sample collection and the non-stressing sampling procedure. In this study, we measured milk cortisol levels prior to grouping the cows to homogenize the groups having the same average milk cortisol levels. This helped us to use these data not only for pre-test evaluation but also as the baseline range. Data from the middle and end of the experiment regarding milk cortisol levels supported the hypothesis and revealed a similar pattern of lower milk cortisol levels in the 12 hG group. This phenomenon could be attributed to the role of the natural environment of the pasture area the grazing cows are exposed to, as previously described [15]. Corroborating the result of this study, in an earlier study, we showed lower hair cortisol levels in cows that grazed in pastures with a natural environment compared to the control indoor system during hot and humid summers [7]. The lower levels of cortisol in the 12 hG group in the middle of the experiment were confirmed by the same measure at the end of the experiment. Unexpectedly, we found higher levels of milk cortisol levels in the 24 hG group compared with the 12 hG and control groups. We actually expected a decrease in the cortisol levels as the time of grazing increased. One hypothesis is that during nighttime rest, the 24 hG group was uneasy throughout the experiment due to lots of rainy days in the pasture area (average rainy days: 3–4). This discomfort among the cows was indicated by the fact that the cows were dirtier, particularly in the udder area, because they lay down in the pasture, which was confirmed by the higher SCC in the same group of cows. Consistent with this claim, Díaz et al. [50] listed various factors that can alter plasma cortisol levels, including but not limited to the management system, such as the type of housing [54] and transport [55], lactation stage [56], and milk yield [57]. Another factor could be the lower feed intake in the 24 hG group compared with the 12 hG group, which can be considered a source of stress, as stated by Gellrich et al. [51] and Fisher et al. [52]. They reported that feed restriction during early lactation, when production is the highest, can cause imbalanced energy levels, leading to activation of the HPA axis [58] and thus increasing cortisol levels in the blood. This is mainly associated with the physiological stage of the cow and its attempt to maintain homeostasis in using body reserves to compensate for the negative energy balance. However, Gellrich et al. [51] reported that the feed intake reduction over the short period in their study did not lead to a stress response involving HPA axis activation and increased cortisol secretion. The cows in this study were still in the high constant lactation period, after the peak, and could be influenced by any stressor, such as lowered feed intake. Overall, the results obtained in milk production and quality in the 24 hG group were expected as well as the high levels of cortisol which is probably associated with a restricted intake as one influential factor. The reasons for lower feed intake in the 24 hG group have been discussed earlier in this article and may explain this phenomenon. Moreover, fighting behavior could be a source of stress in animals [59], however, to what extent the activity of the animals can cause higher activation of HPA activity is not clear. It is because the higher activity levels may increase cortisol concentration in the blood and possibly the milk of animals. Thus, cautions should be taken when interpreting the result of cortisol in active and non-active animals. Bring this to an end, although there are studies in human subjects that fighting behavior in athletes (e.g., martial art) [60] and moderate to high intensity exercise [61] can provoke increases in cortisol concentration, however, further studies in domesticated animals (particularly during grazing) are suggested to investigate the effect of activity level on cortisol concentration.

## 5. Conclusions

Our findings indicate that a 12 h grazing allowance in addition to a conventional confined system, herein the free-stall system along with the TMR-feed-based system, is preferable to only confined management or 24 h grazing allowance with respect to a higher favorable milk fatty acid and lower cortisol levels without adversely affecting milk yield. In this study, the assumption of linear stress reduction with increased grazing time was refuted because of the higher milk cortisol levels observed in the 24 hG group. Thus, grazing is an important influencing factor that can improve animal welfare in addition to other factors such as the quality of management (e.g., use of shade in the pasture area for rainy days; use of smart farming protocols, including using cameras to observe cows in the pasture; providing enriched feed in the pasture to prevent DMI reduction, etc.). Regarding lowering the cost of dairy production systems, which is supported by efficient pasture use in diets, a 12 h grazing allowance in addition to the confined system is suggested to provide the most efficient management system. Grazing dairy cows for 24 h, although economically beneficial, should be performed with cautious or better management practice (e.g., providing TMR or additional concentrate in the grazing area) to avoid low DMI and milk yield and impaired compositions. Further study on grazing allowances for varying amounts of time is needed to identify the most suitable grazing time supporting the highest productivity, in terms of both milk enrichment and economics. Based on the findings of this study, we also suggest further study to find possible relationship between subclinical mastitis, lactose, and grazing pasture allowance.

## Figures and Tables

**Table 1:** Average days in milk, milk yield, body weight, and parity of dairy cows at the beginning of the experiment (*n* = 7).

Item	Control	12 hG	24 hG
Days in milk	110.5 ± 12.5	121.5 ± 20.1	117.6 ± 18.7
Milk yield (kg)	33.5 ± 1.9	32.0 ± 2.5	31.8 ± 3.1
Body weight (kg)	595 ± 27.5	575 ± 21.5	600 ± 31.5
Parity	2	2	2

12 hG: 12 h grazing; 24 hG: 24 h grazing.

**Table 2:** Chemical compositions of the total mixed ration fed to dairy cows during the experiment.

Item	TMR	Concentrate
Dry matter	70.1 ± 35	92.5 ± 1.51
Ash	8.3 ± 1.3	2.9 ± 0.33
Crude protein	15.5 ± 1.2	12.9 ± 0.53
Neutral detergent fiber	45.3 ± 2.7	13.7 ± 2.37
Acid detergent fiber	23.9 ± 2.1	3.7 ± 0.35

**Table 3:** Chemical compositions of the pastures during the experiment.

Item	Sample 1	Sample 2	Sample 3	Sample 4	Average
	**% of DM**				
Ash	7.3	9.5	6.5	8.7	7.9 ± 0.5
Crude protein	13.1	11.5	11.7	10.5	11.7 ± 0.5
Neutral detergent fiber	50.3	53.5	51.7	49.1	51.2 ± 1.3
Acid detergent fiber	30.1	29.9	31.5	27.9	30.0 ± 1.5

**Table 4:** Dry matter intake, milk yield, and milk composition of dairy cows according to treatment group.

Item	Treatment Groups			SEM	Significance Level
	Control	12 hG	24 hG		
DMI, kg/d	21.69 ± 0.83 ^a^	20.85 ± 1.15 ^ab^	16.83 ± 1.41 ^c^	1.013	*
Yield, kg/d					
Actual milk	32.35 ± 1.23 ^a^	29.33 ± 1.55 ^ab^	20.13 ± 2.05 ^c^	1.631	**
3.5% FCM	32.73 ± 1.23 ^a^	30.65 ± 1.43 ^ab^	23.15 ± 1.71 ^c^	1.193	*
Milk composition, %					
Fat	3.57 ± 0.08 ^c^	3.95 ± 0.11 ^b^	4.15 ± 0.17 ^a^	0.130	***
Protein	3.11 ± 0.05 ^ab^	3.35 ± 0.06 ^a^	2.95 ± 0.07 ^c^	0.053	**
Lactose	4.21 ± 0.13 ^ab^	4.39 ± 0.11 ^a^	4.05 ± 0.11 ^c^	0.031	*
SCC (×1000 cells/mL)	120.80 ± 16.13 ^c^	165.30 ± 21.33 ^b^	205.30 ± 20.81 ^a^	19.501	*

* *p* < 0.05; ** *p* < 0.01; *** *p* < 0.001. FCM: fat-corrected milk; SCC: somatic cell count. Control: non-grazing group; 12 hG: 12 h grazing group; 24 hG: 24 h grazing group. ^a,b,c^ Values within a row with different superscripts differ significantly.

**Table 5:** Milk fatty acid composition in dairy cows according to treatment group.

Item	Treatment Groups			SEM ^3^	Significance Level
	Control	12 hG	24 hG		
Total fatty acids (g/100 mL)					
SFA	84.49 ± 1.06 ^a^	78.13 ± 1.07 ^b^	80.30 ± 1.01 ^b^	1.012	*
MUFA	10.15 ± 0.81 ^b^	13.33 ± 0.51 ^a^	13.63 ± 0.45 ^a^	1.321	*
PUFA	6.36 ± 0.83 ^b^	9.54 ± 0.53 ^a^	6.07 ± 0.67 ^b^	1.125	*
Total UFA	16.51 ± 1.01 ^c^	21.87 ± 1.12 ^a^	19.70 ± 1.03 ^b^	1.027	*
SFA/UFA	5.11 ± 0.11 ^a^	3.57 ± 0.13 ^b^	4.07 ± 0.12 ^b^	0.582	**
Fatty acids (mg/100 g)					
Linoleic acid	2.32 ± 0.02 ^c^	2.97 ± 0.01 ^ab^	3.13 ± 0.03 ^a^	0.161	*
γ-linolenic acid	1.13 ± 0.10	0.93 ±0.08	1.19 ±0.11	0.183	ns
Arachidonic acid	3.33 ± 0.18	2.57 ± 0.08	2.41 ± 0.06	0.427	ns
Total ω-6	7.013	6.923	6.75	0.513	ns
α-linolenic acid	0.21 ± 0.03 ^b^	0.49 ±0.03 ^a^	0.37 ± 0.03 ^b^	0.051	***
EPA	0.86 ± 0.05^b^	1.03 ± 0.03 ^a^	0.73 ± 0.05 ^ab^	0.041	**
DHA	0.43 ± 0.07 ^b^	0.81 ± 0.03 ^a^	0.55 ± 0.05 ^ab^	0.027	**
Total ω-3	1.51 ^b^	2.38 ^a^	1.68 ^b^	0.123	**

* *p* < 0.05; ** *p* < 0.01; *** *p* < 0.001. SFA: saturated fatty acid; MUFA: mono-unsaturated fatty acid; PUFA: poly-unsaturated fatty acid; UFA: unsaturated fatty acid; EPA: eicosapentaenoic acid; DHA: docosahexaenoic acid. Control: non-grazing group; 12 hG: 12 h grazing group; 24 hG: 24 h grazing group. ^a,b,c^ Values within a row with different superscripts differ significantly.

## Data Availability

Data are available upon a reasonable request.
